# Disaggregating medical costs due to dementia

**DOI:** 10.1002/alz70860_101357

**Published:** 2025-12-23

**Authors:** Johanna Thunell, Geoffrey Joyce, Bryan Tysinger, Hanke Heun‐Johnson, Duncan Ermini Leaf, Mireille Jacobson, Sidra Haye, Manying Cui, Julie M Zissimopoulos

**Affiliations:** ^1^ University of Southern California, Los Angeles, CA, USA.

## Abstract

**Background:**

Medical care costs of high for persons living with dementia (PLWD) and are particularly high in the year of diagnosis, but remain higher than those without dementia thereafter. In part, this is due to differences in type and amount of health care used and this may vary by sociodemographic and economic background and/or may be due to differences in comorbidities and their management. There is a gap in understanding of the heterogeneity in medical costs due to dementia and the role of comorbid conditions in these costs.

**Method:**

In this study, we use Medicare Parts A, B, and D claims from 2006 to 2020 from 100% traditional Medicare beneficiaries to estimate medical costs among diverse PLWD. We used optimal propensity score matching to match PWLD to similar persons without dementia, using age, sex, race/ethnicity, six comorbid conditions, and Charlson Comorbidity Index. We quantified medical costs due to dementia, by type of medical care and across region, sex, race/ethnicity, and among PLWD with a high‐cost comorbid condition of diabetes. We report costs for PLWD and matched persons without dementia.

**Result:**

In 2020, average medical costs across all settings were $35,828 among PLWD, compared to $29,752 among matched persons without dementia. Nearly half the $6,077 difference in overall costs was due to inpatient costs ($2,943). Cost differences attributable to dementia were largest among Asians ($8,931) and peaked at ages 65‐70 ($7,992). Prevalence of diabetes among PLWD was 38.3% compared to 27.6% among persons who never developed dementia. Average medical costs for PLWD with diabetes were over 2 times higher for PLWD ($46,772), compared those without dementia ($21,140).

**Conclusion:**

This research will be the first to present nationally‐representative estimates of medical costs attributable to dementia by region, sex, race and comorbid status. Our final set of analyses will further disentangle costs across additional patient subgroups, settings, and time to ascertain when, where, and for whom cost of dementia is highest. Results will inform comprehensive estimates of cost of dementia using the USCDM and may have clinical implications for management of comorbid conditions in PLWD.

